# Predicting Lung Health Trajectories for Survivors of Preterm Birth

**DOI:** 10.3389/fped.2020.00318

**Published:** 2020-06-19

**Authors:** James T. D. Gibbons, Andrew C. Wilson, Shannon J. Simpson

**Affiliations:** ^1^Telethon Kids Institute, Perth, WA, Australia; ^2^School of Physiotherapy and Exercise Science, Curtin University, Perth, WA, Australia; ^3^Department of Respiratory and Sleep Medicine, Perth Children's Hospital, Nedlands, WA, Australia

**Keywords:** preterm, bronchopulmonary dysplasia, lung function, infant, lung function trajectory

## Abstract

Rates of preterm birth (<37 weeks of gestation) are increasing worldwide. Improved perinatal care has markedly increased survival of very (<32 weeks gestation) and extremely (<28 weeks gestation) preterm infants, however, long term respiratory sequalae are common among survivors. Importantly, individual's lung function trajectories are determined early in life and tend to track over the life course. Preterm infants are impacted by antenatal, postnatal and early life perturbations to normal lung growth and development, potentially resulting in significant shifts from the “normal” lung function trajectory. This review summarizes what is currently known about the long-term lung function trajectories in survivors of preterm birth. Further, this review highlights how antenatal, perinatal and early life factors are likely to contribute to individual lung health trajectories across the life course.

## Introduction

Rates of preterm birth are increasing. Currently, around 11% (~15 million) of global births are preterm (<37 weeks of gestation) and over 2 million of these annual births are very preterm (<32 weeks gestation) (1). Enhanced perinatal care, including antenatal steroids, postnatal surfactant and improved respiratory management, have markedly improved survival outcomes for all preterm infants but particularly for those born <32 weeks gestation. However, long term sequalae are common in very preterm survivors, particularly of the lungs.

Recent studies of lung health throughout childhood, adolescence and early-adulthood have ignited debate that survivors of very preterm birth are destined for chronic obstructive pulmonary disease (COPD), a debilitating and life threatening lung disease with few effective treatments (2–4). Given the increased incidence of preterm birth, and the increased survival of preterm babies, it is also likely that the global burden of preterm-associated lung disease is set to rise. Consequently, it is critical that we work toward understanding the implications for lung health across the life course in survivors of preterm birth. This review aims to explore what is currently known about the long-term lung function trajectories in survivors of preterm birth and to investigate the factors contributing to individual lung health trajectories.

## Bronchopulmonary Dysplasia

Bronchopulmonary dysplasia was first described 50 years ago in infants with a mean gestational age (GA) of 34 weeks and prolonged exposures to mechanical ventilation and high concentrations of oxygen (O_2_) (5). The modern definition of BPD is the requirement for supplemental oxygen for at least 28 days (6), with severity assessed by level of supplemental oxygen and/or continued respiratory support required at 36 weeks postmenstrual age. Improvements in neonatal critical care such as the introduction of postnatal surfactant, routine use of antenatal maternal corticosteroids for fetal lung maturation, and the development of non-invasive ventilation strategies over recent decades mean that BPD is rarely seen today in infants born after 32 weeks gestation (7). Consequently, the clinical and pathological characteristics of lung health in survivors of preterm birth and bronchopulmonary dysplasia have changed over time. The contemporary disease, termed ‘new BPD', has less prominent airway pathology and is dominated by peripheral lung abnormalities including failed alveolarization with a decreased number of large and simplified alveoli, and abnormal pulmonary vascular development (8). Very preterm infants surviving the neonatal period have a significant burden of lung disease in the first years of life: increased respiratory symptoms, increased health care utilization and increased hospital admissions are reported consistently (9). This burden rises further with increasing prematurity and the co-existence of BPD. Contemporary bronchopulmonary dysplasia is a risk factor for long-term adverse outcomes of prematurity. Survivors of preterm birth have been shown to have impaired lung health beyond the preterm years, with persistent and burdensome respiratory symptoms and abnormal lung function; the underlying pathology of which remains relatively unknown beyond infancy. There remains no way to predict during the neonatal period which infants will have ongoing or lifelong breathing problems.

## Lung Function Trajectories and Course Modifiers

**Figure 1:**
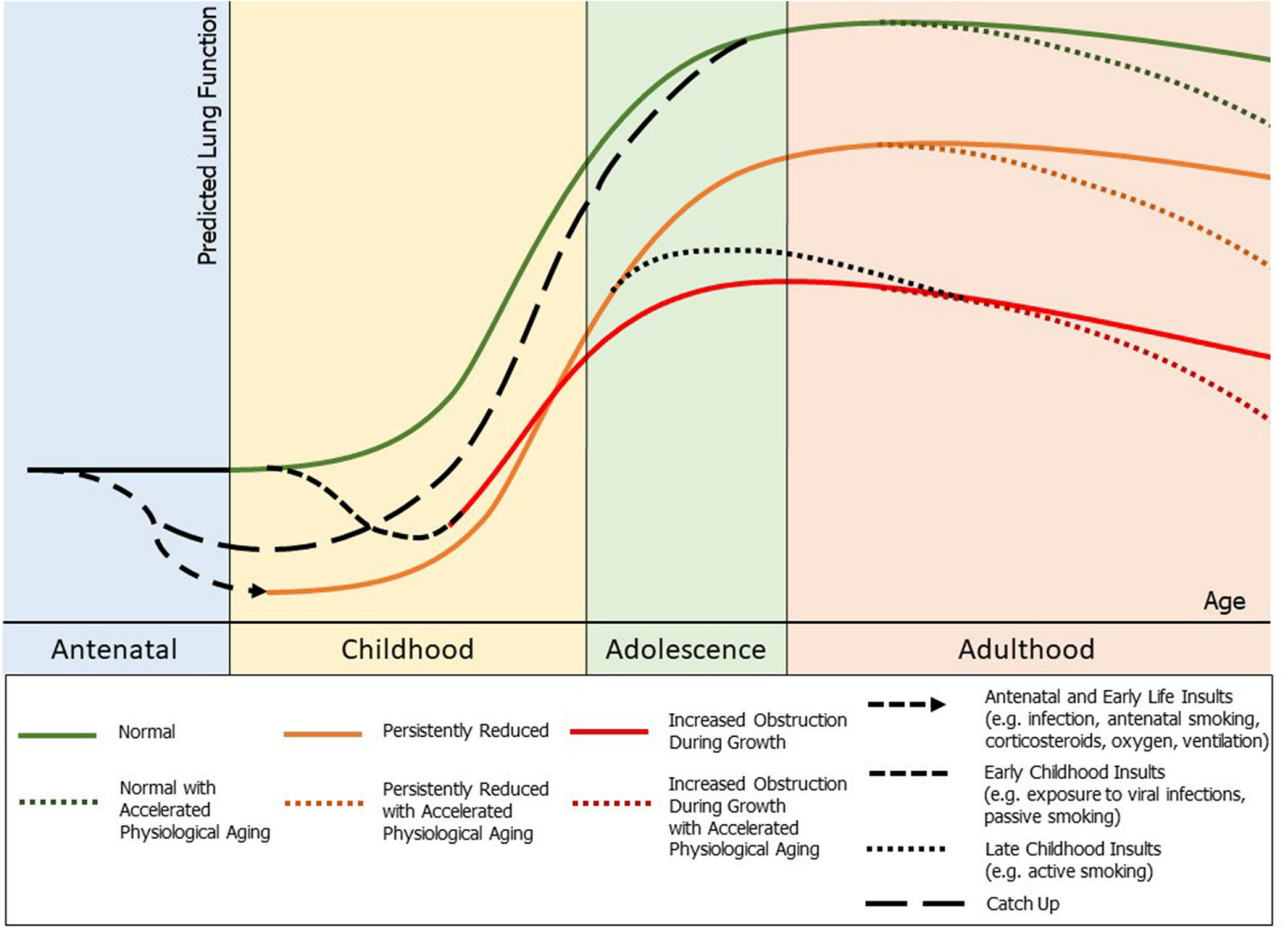
Outline of potential lung function trajectories and lung function perturbations throughout life: normal (green), persistently reduced lung function (orange), and early decline in lung function with increased obstruction during growth (red), with a potential for accelerated physiological aging in adulthood. Potential insults to lung health and their possible impact on lung function throughout life marked. Also noted is a potential for “catch up” growth to normal lung function which may occur during childhood and adolescence.

In healthy individuals, lung growth and development, measured by lung function, follows a distinct pattern (or trajectory) over the life course (Figure 1, green). This trajectory comprises three discrete phases: a growth phase (birth to early adulthood), a short plateau phase, and a decline phase (physiological lung aging). Birth cohort studies show that lung function tracks over the life course. Importantly, low lung function at birth (and early childhood) establishes a lifelong trajectory of low lung function, and is independently associated with chronic lung disease (10). Additionally, lung function trajectories are affected by perturbations to the system during critical windows of lung growth and development, resulting in shifts from the “normal” trajectory.

Preterm birth (<37 weeks of gestation) represents a significant perturbation to lung development. Further, the *in-utero* environment of preterm infants is often compromised prior to a preterm delivery (Figure 1, antenatal insults). Once delivered, the preterm infant must commence (often ineffective) gas exchange with incompletely developed lungs. Therefore, many preterm infants receive injurious, but lifesaving, treatments (such as positive pressure ventilation) during a critical developmental window. Furthermore, early or mid-late childhood factors such as exposure to viral infections or cigarette smoke (passive or active) can cause insults to the developing lungs and have a negative impact on long term lung function (2, 11) (Figure 1, childhood insults). Understanding lung function trajectories for survivors of preterm birth is critical since reduced lung function is a major cause of morbidity and a significant predictor of all-cause mortality, even in the general population (12). Indeed, a recent national cohort study from Sweden showed that survivors of preterm birth have markedly increased risk of death throughout life with lung health a major contributor (13).

There are only limited longitudinal studies of survivors of very preterm birth which provide data on lung function, and they offer conflicting evidence. Persistent reductions in lung function (forced expiratory flows and volumes by spirometry) have been reported in a few studies (Figure 1, orange). A cohort of 17 survivors of BPD were followed up prospectively by Filippone et al. (14) and showed persistently reduced lung function compared with a control group when followed up at 2, 9, and 15 years, and no improvement in lung function trajectories throughout this time period. Similarly, Tunqvist et al. (15) showed in a Swedish cohort study that survivors of moderate-to-late preterm birth have reduced function at 8 and 16 years, with no catch up growth reported. Vollsaeter et al. (16) also followed two cohorts of survivors of preterm birth <28 weeks or <1000 g, one from 10 to 18 years of age (surfactant era), and one from 18 to 25 years of age (pre-surfactant era), with both groups showing significant reductions in forced expiratory flows and volumes extending into adulthood, which did not improve with time. There is also concern that some cohorts of preterm children may have early decline in lung function trajectories (Figure 1, red). Simpson et al. (17) followed a cohort of children born ≤32 weeks gestation born in the surfactant era longitudinally between early childhood and mid-childhood (4–12 years) and demonstrated a decline in lung function (increased airway obstruction by spirometry) and worsening peripheral respiratory mechanics (as assessed with the forced oscillation technique) between these periods. Doyle et al. (18) also demonstrated a decline in lung function with signs of increased airway obstruction in children between 8 and 18 years of age. Furthermore, persistent reductions in the small airway function of survivors of preterm birth, tracking from 1 year corrected age to 11–14 years of age, has recently been suggested by Lo et al. (19). Given that survivors of very preterm birth in the surfactant era are just now reaching their 30's, we are yet to determine if accelerated physiological aging will be observed as a result of preterm birth itself, or in addition to the known consequences of adult exposures (tobacco smoke, occupational exposures, etc.) (Figure 1, accelerated physiological aging).

## Prematurity Related Risk Factors Affecting Lung Function Trajectories

### Antenatal Factors

#### Antenatal Corticosteroid Use

Maternally administered corticosteroids have a well-established role in reducing the rates of many adverse outcomes associated with preterm birth including perinatal death, RDS, need for mechanical ventilation, intraventricular hemorrhage, necrotizing enterocolitis, and systemic infections in the first 48 h of life (20, 21). When the preterm fetus is exposed to antenatal corticosteroids there is an accelerated maturation of the lung and stimulation of surfactant production from type II epithelial cells, providing a more functionally mature lung better suited to gas exchange. Animal studies have shown that glucocorticoids can also acutely change lung structure, thinning the mesenchyma and decreasing alveolar septation, which improves lung compliance and gas exchange in the short term, however may also have clinical implications with decreased alveolar septation being a part of the primary disease process of BPD (22).

Despite much literature on the short-term benefits of antenatal corticosteroids, there is limited evidence on their long-term impact on lung health. Doyle et al. (23) followed up an cohort of low birth weight children (<1,501 g), born between 1980 and 1982, at 14 years of age who were exposed to antenatal corticosteroids non-randomly and found those exposed were significantly taller and had better cognitive function, though they had no differences in lung function at a single timepoint. Similarly, no differences in lung function were reported by Dalzeil et al. (24) when following up survivors of preterm birth born been 24 and 36 weeks gestation at 30 years of age given betamethasone as part of an RCT in the pre-surfactant era. However, Simpson et al. (See supplementary file) reported bigger declines in lung function trajectories over time in those exposed to antenatal steroids compared to those that were not exposed (17). Given the routine use of antenatal corticosteroids in modern antenatal care for their benefits in treating the acute complications of preterm birth it will be difficult, but necessary, to determine their long term impact on lung function.

#### Chorioamnionitis and Fetal Inflammatory Response

Acute and chronic chorioamnionitis are commonly reported lesions in the placenta after spontaneous preterm birth (25, 26). It has been hypothesized that chorioamnionitis is associated with increased pulmonary inflammation which may result in BPD and impaired lung health (27). However, it has also been suggested that pro-inflammatory factors associated with chorioamnionitis may be protective against RDS and BPD by promoting early lung maturation (28, 29).

A recent meta-analysis by Sarno et al. (30) explored these theories and found that, after adjusting for the effects of gestational age, histologically confirmed chorioamnionitis was associated with a reduced risk of RDS and did not affect the development of BPD. Ballard et al. (31) supported these findings in a 25 year cohort study of children born ≤32 weeks gestation and birthweight <1,500 g showing that while neonatal sepsis was associated with a diagnosis of moderate or severe BPD, a histological diagnosis of chorioamnionitis was not. Lahra et al. (29) found evidence that a fetal inflammatory response may be protective for chronic lung disease in a cohort study of 798 infants born in Sydney, Australia. Umbilical vasculitis, as a marker of a fetal inflammatory response, showed a significant protective effect against the development of chronic lung disease. Similar findings have been made in a case-control study of preterm infants by Van Marter et al., with histological chorioamnionitis in the absence of postnatal sepsis and ventilation was protective against BPD. Meanwhile, a study of infants born <33 weeks gestation by Prendergast et al. (32) who all received antenatal steroids and postnatal surfactant found no association between chorioamnionitis and BPD, nor any significant differences in lung function during the perinatal period or at 36 weeks postmenstrual age.

There is limited evidence on how chorioamnionitis affects long-term lung function. Chorioamnionitis has been directly associated with reduced long term lung function a study of preterm infants (<37 weeks gestation) by Jones et al. (33) who found that increasing severity of histological chorioamnionitis was associated with a significant downward trend in lung function measured in the first year of life after 40 weeks postconceptional age. At present there do not appear to be any further studies investigating the long-term effects of chorioamnionitis on lung function trajectories.

#### Antenatal Smoking

Maternal smoking during pregnancy is a significant modifiable risk factor associated with substantial pregnancy related morbidity and mortality, and has been identified as a direct risk factor for inducing preterm birth (34). Antenatal smoking exposure has also been associated with increased respiratory symptoms and lung function abnormalities throughout later life (35–38).

Animal models may provide some indication as to the underlying mechanisms of the harmful effects of antenatal nicotine exposure. Rodent studies have shown that nicotine exposure can decrease maximal expiratory flow by stimulating epithelial cell growth and lung branching, resulting in more tortuous airways (39) and a reduction in elastin synthesis with fewer but larger alveoli of similar appearance to emphysema (40). Antenatal nicotine exposure in guinea pigs causes fewer alveolar attachment points and increased airway responsiveness (41). While in a study of rhesus monkeys by Sekhon et al. (42), antenatal nicotine exposure caused reduced fetal lung growth and reductions in forced expiratory volumes and flows.

Studies in human populations have showed similar effects to those seen in the animal studies. The detrimental effects of antenatal smoking have been well documented (35, 36). Reductions in FEV1 and mid-expiratory flows have been reported by Moshammer et al. (43). Gilliland et al. (44) also found that antenatal smoking was associated with a reduction in FEV_1_/FVC and FEV_25−75_ in children and adolescents aged from 7 to 18 years. Reductions in FEV_1_ and FEV_25−75_ appear to persist into early adulthood in a study by Hayatbakhsh et al. (38), indicating that the lung function abnormalities present in childhood and adolescence are not reversible. Antenatal smoking exposure has also been associated with an increased risk of wheeze and asthma diagnosis during childhood and adolescence (44–47).

There is limited data reflecting how the combination of being born preterm and antenatal smoking exposure may affect long-term lung health. Robison et al. (48) found evidence of an interaction between prematurity and antenatal smoking causing an increased risk of wheeze in a cohort study of 1,448 children. Similar evidence was found by Kotecha et al. (49) in a recent analysis of data from the Millennium Cohort Study, with an increased risk of wheeze in preterm children exposed to antenatal smoking. This may suggest that survivors of preterm birth with a history of antenatal smoking exposure are at risk of an earlier decline in their lung function trajectories, however more longitudinal studies are required to quantify this.

### Perinatal and Early Life Factors

#### Gestational Age

Multiple studies have shown that preterm children born at a lower GA have more significant reductions in lung function and a greater impact on overall lung health than those born at a later GA (17, 18, 50–53). There is an increased risk of exposure to almost all subsequently discussed risk factors, the preterm lungs are exposed to numerous insults such as increased oxidative stress and inflammation at an earlier developmental stage (54). This is reflected in the limited pathological studies of the contemporary lung disease associated with preterm birth showing an enlarged and simplified alveolar structure with dysmorphic pulmonary vascular growth (55).

Considerable effort is made to prevent preterm birth where possible, with significant advances in maternal care being made in recent history, and multifaceted public health programs targeting multiple strategies to reduce the overall rate of preterm birth proving effective (56, 57). While the gold standard of reducing lung disease associated with preterm birth will remain reducing the overall rates of preterm birth, with continual improvement in resuscitation techniques and neonatal care it is likely that rates of preterm birth will continue to rise requiring greater knowledge on what other risk factors can be identified and modified to improve short and long term outcomes.

#### Exposure to Supplemental Oxygen During the Neonatal Period

Respiratory distress syndrome (RDS) occurs early in the lungs of preterm infants as a result of damage to the airway epithelium caused by a lack of surfactant production in an immature airway, resulting in the activation of inflammatory processes (58). A consequence of this is often an early and increasing oxygen requirement. While often essential therapy to avoid systemic hypoxia, oxygen administration as a neonate is also a likely cause of ongoing lung pathology through mechanisms such as increased oxidative stress and pro-inflammatory mechanisms which continue to occur during the neonatal period (54).

Animal models provide evidence on the longitudinal effects of oxygen exposure in prematurity which is not yet available in humans. In mouse studies performed by Yee et al. (59), newborn mice were exposed to oxygen concentrations ranging between 21 and 100% until postnatal day 4 before being returned to room air until adulthood at 8 weeks of age. At 8 weeks of age, a simplified alveolar structure was noted in the mice exposed to 60 and 80% oxygen, and findings were noticeably worse when the mice exposed to 100% oxygen. Similarly, lung compliance was increased in mice exposed to 60 and 80% oxygen, and further increased in those exposed to 100% oxygen. This study would suggest not only that there is a dose effect of oxygen, but also that the damage from exposure to oxygen exposure shortly after birth is not corrected in later life.

Lung function studies on survivors of preterm birth provide further evidence that oxygen independently is associated with long term damage to the lung. Simpson et al. (60) showed that each additional week of neonatal oxygen exposure as associated with further decrement in forced expiratory volume in 1 s (FEV_1_), forced vital capacity (FVC), forced expiratory flow at 25–75% of FVC (FEF_25−75_,), and the elastic properties of the lung (respiratory system reactance at 8 Hz) in survivors of preterm birth at 9 to 11 years of age, indicating there may be ongoing damage to the airways and peripheral lung (60). Further, the same group demonstrated declines in these measures over time in children with increased oxygen exposure (17). An obstructive picture on spirometry measurements also been reflected in other cohort studies investigating survivors of preterm birth (18, 61, 62). Further to this, Stevens et al. (63) showed evidence which would suggest that in addition to duration of oxygen exposure, an increasing total cumulative dosage of oxygen (measured by an area under the curve analysis of oxygen concentration and time on oxygen) had detrimental effects causing increasing symptomatic airway dysfunction at 1 year of age.

Oxygen has also been suggested to have a direct impact on long term structural effects of the lung in a study by Aukland et al. (64) investigating survivor of preterm birth born in both a pre-surfactant era (1982–85) and surfactant era (1991–2). Prolonged oxygen exposure was associated with significantly more lung parenchymal abnormalities in both cohorts on computerized tomography (CT) scans, where no other measured factors were noted to have a significant impact in regression analysis.

An increased risk of bronchial hyper-responsiveness has been associated with prolonged neonatal oxygen exposure in survivors of preterm birth (65, 66). In a study of 12-year old survivors of preterm birth born between 1987 and 1978 with a birth weight ≤1500 g, Mai et al. (65) found oxygen exposure ≥ 9 days was associated with an increased risk of asthma diagnosis on multivariate analysis. Similar findings were made by Halvorsen et al. (66) investigating two groups of survivors of preterm birth at ~11 and 18 years of age, with a significant association between airway hyper-responsiveness (as measured through a methacholine challenge) and days of oxygen treatment as a neonate. A limitation of these studies is that they come from the pre-surfactant era of neonatal care at a time where the modern practices of minimizing oxygen exposure were not used. Currently no longitudinal studies in the contemporary era of preterm birth have adequately examined the link between oxygen exposure and airway hyper-responsiveness, however bronchial hyper-responsiveness does continue to be seen in contemporary survivors of preterm birth. Early studies suggested wheezing in later life in survivors of preterm birth was due to asthma, however more recent evidence suggests that the underlying mechanisms of bronchial hyper-responsiveness in survivors of preterm birth are different to those seen in asthmatics, indicating a different pathological process is responsible for wheeze in this population (67).

While the above studies may suggest that oxygen exposure is toxic to infants, it must be interpreted with caution as supplemental oxygen is often also essential in neonatal care. This has been highlighted by the results of the BOOST II trial where preterm neonates randomized to receive supplemental oxygen targeting saturations at 85 to 89% had a significantly higher mortality rate than those who targeted saturations of 90–94% (68).

#### Effects of Ventilation Technique and Duration

Invasive or non-invasive mechanical ventilation is frequently required by preterm neonates to support gas exchange while they are affected by RDS, however it can have detrimental effects to lung health as the preterm lung is incredibly vulnerable to injury. The preterm lung with RDS has lower relative lung volumes to overall size, and lower pressures required to achieve lung opening and total lung capacity (58). As such, the preterm lung is susceptible to damage from excessive tidal volumes (volutrauma) or pressures (barotrauma), which further increases the inflammatory processes at play, increasing pulmonary oedema, and reducing the ability to exchange gas. In addition, the immature and surfactant deficient lung has non-uniform inflation which can result in focal overdistension, meaning that lung injury can occur in the absence of high volumes and pressures.

There has been an increasing shift away from endotracheal intubation to less invasive forms of ventilation such as nasal continuous positive airway pressure (CPAP) and heated humidified high flow nasal cannula (HFNC) (69). This move has been driven by an effort to avoid the more detrimental effects endotracheal intubation such as barotrauma and volutrauma, and has been shown to have a small but significant effect on reducing the risk of developing BPD, although with unclear impacts on long term respiratory function (70). The duration of endotracheal intubation has been found to be negatively associated with FRC when measured at 15–18 months corrected age suggesting that prolonged intubation leads to long term airway disease (71). Interestingly, Doyle et al. showed that despite increased use of less invasive ventilation over time, the duration of oxygen therapy and the rate of oxygen dependence at 36 weeks increased, and airflows at 8 years of age were worse in children born in 2005 than in earlier periods (72).

Improvements in ventilator technology have allowed for a shift from traditional pressure limited ventilation (PLV) to volume-targeted ventilation (VTV) strategies with the aim of reducing volutrauma to the lungs caused by inconsistent tidal volumes being delivered to the lungs (73). The current evidence suggests that neonates ventilated using VTV require a shorter duration of mechanical ventilation, reduced rates of pneumothorax, and less neurological sequelae such as intraventricular hemorrhages and periventricular leukomalacia (74). Limited evidence exists, however, to suggest whether the shift toward VTV from PLV will have an impact on long term lung function. A group of 85 survivors of preterm birth born between 24 and 31 weeks gestation who were randomized to either PLV or VTV were followed up Singh et al. (75) at ~2 years of age with a finding of significantly reduced use inhaled medications and a trend toward reduced respiratory symptoms in the PLV group. Currently there are no other studies which provide evidence on the long-term respiratory outcomes of VTV compared with PLV, and whether the theoretical advantages of reduced volutrauma as a neonate translate into long term improvements in lung function.

There has been interest in HFNC as an alternative form of non-invasive respiratory to CPAP over the last two decades, predominantly because it is easier to set up and maintain, is more comfortable for infants, and is preferred by nurses and parents (76–79). Initial studies suggested that when used as a primary respiratory support after birth there were no differences in the rates death or chronic lung disease (80). Recent evidence, however, has suggested HFNC is an inferior as a mode of primary respiratory support in preterm infants >28 weeks gestation, or when used as post-extubation respiratory support when compared with CPAP (81, 82). No longitudinal studies have thus far compared the outcomes of those who have used HFNC with those using CPAP, making it difficult to speculate on what effect the use of HFNC may have on long-term lung health.

Over the last two decades there has been increased uptake of high frequency oscillatory ventilation (HFOV) techniques to minimize the effects of barotrauma caused by conventional mechanical ventilation (CMV), though evidence in this field is extremely limited. HFOV delivers small tidal volumes at rapid rates, usually between 10 and 15 Hz (83). HFOV has been shown to reduce BPD in preterm infants, however the long term implications on lung function in the surfactant era remain unclear (83, 84). Conflicting evidence exists as to whether HFOV offers advantages over CMV. A meta-analysis of individual patient data by Cools et al. (85) found HFNC to be equally effective to CMV with regards to relative risk of death, BPD, or serious adverse neurological event, and did not support HFOV as a mode of ventilation for preterm infants. Other studies, however, have suggested HFOV may offer long term advantages to lung health. A randomized control trial in surfactant treated preterm newborns by Gerstmann et al. (86) demonstrated evidence that children treated with CMV showed decreased peak expiratory flow, increased residual lung volume, and maldistribution of ventilation when reviewed at 6 years of age compared with those using HFOV. FEV1/FVC values were lower than expected in the HFOV group, although not significantly different to those treated with CMV. The United Kingdom Oscillation Study reported by Zivanovic et al. (87) followed up survivors of preterm birth <29 weeks gestation who were initially randomized to HFOV or CV through to 11-14 years of age. This study found significant differences in lung function with FEV1, FEF25, FEF50, FEV1:FVC radio, PEF, DLCO, vital capacity, and respiratory resistance all favoring the group treated with HFOV. These differences were, however, relatively small (on average 0.3 SD), with the clinical significance of such changes unclear. Further study of this population would be beneficial to determine if HFOV improves lung function trajectories throughout life.

#### Postnatal Corticosteroid Use

The use of postnatal corticosteroids in the management of preterm infants with BPD has varied significantly over the last several decades (88). Initially considered the logical option for managing the inflammatory effects of BPD, concerns of significant neurotoxic side effects have resulted in significant reduction in systemic corticosteroid use in many centers (89). Doyle et al. showed evidence of short term respiratory benefits from systemic corticosteroids when given at <8 days (90) and >7 days (91) but the risks of long term neurodevelopmental side effects from postnatal corticosteroids were considered to outweigh any potential benefits.

The long-term respiratory outcomes associated with postnatal corticosteroids have not been well defined, with few studies specifically commenting on this area. Some evidence exists from Nixon et al. (92) who followed up a cohort of children with a birthweight <1501 g who were given a 42-day postnatal dexamethasone course in order to attempt to reduce ventilator dependency. When pulmonary function was assessed between 8 and 11 years of age, a significantly greater proportion of children in the placebo group had an FEV1 below the 5th percentile when compared with the treatment group, although the difference between groups when directly compared was not statistically significant (P value of 0.08). Another randomized control trial comparing postnatal dexamethasone given between 2 and 12 weeks of age in children with neonatal chronic lung disease also provided some longitudinal follow up (93). This study showed no significant difference lung function or respiratory outcomes between groups, though lung function was noted to be impaired in both groups. Meanwhile, postnatal steroid use was associated with more severe chronic lung disease in an observational study by Gage et al. (94) of infants between 4 and 9 months corrected gestational age with a history of preterm birth born at <1500 g and <30 weeks gestational age. Aukland et al. (64) also noted an association between increasing lung pathology on computerized tomography (CT) scans and postnatal corticosteroid use. An increased rate of asthma in 8 to 11 year old survivors of preterm birth (born ≤ 36 weeks gestation) has also been linked with postnatal corticosteroid use in an observational study by Grischkan et al. (95).

There is emerging evidence that early administration of inhaled corticosteroids may reduce the risk of chronic lung disease in children, without many of the significant side effects of systemic corticosteroids. A Cochrane review by Shah et al. (34) suggested that early administration of inhaled corticosteroids reduced the combined risk of death and chronic lung disease at 36 weeks post menstrual age, though no significant reduction in chronic lung disease in isolation was noted. More recently, Tukova et al. (96) found that in a cohort of extremely preterm children (23 to 27 weeks gestation), randomized to receive inhaled budesonide for at least the first two weeks of life as part of the Neonatal European Study of Inhaled Steroids, lung function was not significantly different when measured at 5 to 7 years, however there was a significantly reduced rate of symptoms associated with chronic lung disease. The long-term outcomes of early inhaled corticosteroid use remain unknown. The long-term effects of postnatal corticosteroids on lung function should remain an area of interest, particularly in the event that practice was to change to favor their use again in certain cohorts.

#### Effects of Surfactant

Like antenatal steroids, surfactant administration to preterm infants revolutionized neonatal care due to its ability to prevent and treat respiratory distress syndrome by improving pulmonary compliance and reducing surface tension in newborns with immature type II pneumocytes (97, 98). Unfortunately, while the short term benefits of surfactant are clear, the long term benefits or risks of surfactant therapy to lung health has been less clearly defined as follow up of the original studies was predominantly was reported in only the first 3 years of life (99).

The current evidence does appear to support the notion that childhood lung function in survivors of preterm birth in the post-surfactant era has improved when compared with survivors of the pre-surfactant era. A small study by Pelkonen et al. (100) followed up an early group of 40 children at between 7 and 12 years of age who were randomized to surfactant treatment in an earlier randomized control trial as either a prophylactic or rescue medication, and found higher peak expiratory flow and FVC values in those treated with surfactant in either group compared with those who weren't. Vollsaeter et al. (101), meanwhile, found significant improvements in FEV1 z-scores in 11 year old survivors of preterm birth born in the surfactant era (1999–2000) when compared to survivors of preterm birth born before surfactant use was mainstream (1991–92), and that after adjusting for the use of surfactant and antenatal steroids, these improvements were negated, suggesting that increasing use of these modalities may partly explain this improvement. The Victorian Infant Collaborative Study Group (VICS), compared lung function in survivors of preterm birth born <28 weeks gestation and <1,000 g at birth in both the pre-surfactant (1991–2) and post-surfactant (1997) eras (51). Both cohorts showed similar reductions in lung function when compared with control populations, with no clearly demonstrated improvements in the 1997 cohort which could possibly suggest that surfactant has not assisted long term outcomes. It should be noted, however, that the survival rate of the 1997 cohort was significantly higher and therefore may include more children with worse lung function who may not have survived in the previous era.

An area which warrants monitoring moving into the future is how the long-term outcomes of preterm infants who less invasive surfactant administration (LISA) compare with those who receive surfactant delivered via an endotracheal tube. In this technique, surfactant is delivered to the trachea by a thin catheter, then dispersed to the lungs using nasal CPAP without the need to perform endotracheal intubation (102). There has been increasing interest in LISA over the last decade as it there is increasing evidence that this technique results in reduced rates of mechanical ventilation, BPD, and death (103). Currently, outcomes have not been reported past the early life period, but LISA should remain an area of interest moving forward.

#### Caffeine

Caffeine, a methylxanthine, has become a commonly administered medication for preterm neonates over the last two decades. The Caffeine for Apnea of Prematurity (CAP) trial identified the benefits of caffeine, showing that neonates who were given caffeine between days 3 and 10 of life for apnoea of prematurity had reduced rates of BPD, decreased duration of mechanical ventilation, and improved survival without neurodevelopmental disability at between 18 and 21 months (104, 105). More recent data has also shown improved long term outcomes for children treated with caffeine, with significantly improved expiratory flows at 11 years of age (106).

Timing of caffeine administration has also been an area of recent interest, with evidence suggesting that early caffeine administration (within 2 days of birth) may provide more neurodevelopmental benefits and reduced rates of BPD compared with late caffeine administration (107–110). A comparison of the long-term effects of early and late caffeine administration has yet to be reported, however remains an area of interest in future if it may influence lung function trajectories later in life.

#### Neonatal Sepsis

Children born preterm, particularly very low birth weight infants, are at a significantly increased risk of neonatal sepsis due to immature innate and adaptive immune systems (111). Postnatal sepsis has been shown to be an independent risk factor associated with the development of moderate or severe BPD in preterm infants after controlling for gestational age (31, 112). The effects of chorioamnionitis and intrauterine inflammation discussed earlier should be considered in conjunction with the effects of postnatal sepsis, due to the former often causing the latter.

The previously mentioned study by Lahra et al. (29) which showed a protective effect of umbilical vasculitis against chronic lung disease also showed that neonatal sepsis was associated with a significantly increased risk of chronic lung disease, independent of the effects of umbilical vasculitis. This was particularly evident in those with coagulase-negative staphylococcal bacteraemia. Meanwhile, a cohort study of 5,447 very low birth weight infants by Stoll et al. (113) found that rates of BPD increased from 35 to 62% in the presence of early onset sepsis. These findings were subsequently validated in an Israeli population study of 15,839 infants (114). Late-onset sepsis in preterm infants has also been associated with an increased risk of BPD in a retrospective analysis of infants born <32 weeks in the Canadian Neonatal Network (115).

The long-term impact of postnatal sepsis as a risk factor for altered lung function trajectories throughout life has yet to be determined. Significant advances have been made over the last two decades in the identification of infants at risk of sepsis, early diagnosis, and management strategies (116). It would be expected that, if postnatal sepsis is associated with a deterioration in lung function trajectories, these advances in neonatal care should improve also be associated with improvements in lung health. More study is required, however, to validate this theory.

### Childhood and Early Adulthood Factors

#### Early Life Viruses

Exposure to viral infections in early life has been shown to have the potential to have significant impact on lung function trajectories in healthy individuals, and those with asthma (117–119). As has been made evident in a recent review by Townsi et al. (11) exposure to viral infections in early life by survivors of preterm birth can have a significant impact on short and long term lung health. Early exposure to viral infections has substantial implications with the risk of developing BPD more than two times higher among infants who have detectable respiratory viruses during their birth hospitalization when compared to those without viral infections (120). Preterm infants also have an increased risk of rehospitalization with an acute viral infection within their first year of life approximately three times that of term born infants (121). This risk is significantly higher for preterm infants with BPD who remain oxygen dependent during the first three years of life (122).

Exposure to respiratory syncytial virus (RSV) early in life has also been identified as a significant factor in the developmental of suboptimal lung health and lung function in both term and preterm children. Studies have shown that children exposed to RSV have an increased risk of: late-onset or persistent wheezing phenotypes; a diagnosis of asthma; and impairments in lung function during adolescent or young adult life (117, 123–125). Palivizumab, a humanized monoclonal antibody used to prevent RSV infections, has shown evidence that it may significantly reduce acute and chronic morbidity following RSV infection in preterm infants (126, 127). The use of palivizumab is generally restricted, however, due to the high financial cost of the treatment and uncertainty regarding cost-effectiveness (128, 129).

Ongoing improvements in prevention and treatment of viral infections in this population is needed to minimize the impact that they have on lung health, with potential interventions like an RSV vaccine offering promising views for the future (130).

#### Postnatal Smoking

Maternal smoking and personal smoking have been established as risk factors toward the development of chronic obstructive pulmonary disease (COPD) as an adult, and more rapid declines in lung function trajectories (2, 131). It is reasonable to speculate that children with a history of preterm birth who have already experienced an insult to their lung health may be more predisposed to the harmful effects of smoking than their term counterparts.

At present, no study appears to compare lung function in adults who smoke between those born at term and those born preterm to further explore this. There is evidence, however, that survivors of preterm birth exposed to passive smoke during childhood (parental smoking) (17) and those who smoke during adolescence have a more rapid decline in their lung function trajectories compared with those who don't smoke (18).

#### Inhaled Corticosteroids in Childhood

Very limited evidence exists investigating the use of corticosteroids beyond the neonatal period, with even fewer investigating the effects of inhaled corticosteroids. Yuksel et al. (132) found a group of 18 preterm infants (28 weeks gestation) who had respiratory symptoms at a 10 month follow up had significant reductions in symptoms when treated with a 6 week course of inhaled corticosteroids compared with placebo. Studies done in older children (aged 7 to 13 years) showed no significant improvements in lung function or decrease in symptoms following a course of inhaled corticosteroids, although decreased variability was noted in peak expiratory flows, which may suggest decreased underlying bronchial liability (133, 134). While asthma symptoms, particularly wheeze and cough, have been noted by many in survivors of preterm birth, the mechanisms underpinning this disease may not be eosinophilic in nature, and therefore the role of inhaled corticosteroids remains unknown in this population, and larger randomized controlled trials are required to elucidate this (67). It remains unknown what effect treatment with inhaled corticosteroids through childhood has on longitudinal lung function, and how it may impact on lung function trajectories.

## Interplay Between Risk Factors Impacting on Lung Function Trajectories

While we have considered each of the identified risk factors for reduced lung function in relative isolation during this review, it is important to consider how these factors will interact with each other to have a potentially cumulative impact on lung health. For instance, a child born at 24 weeks gestation is much more likely to require intensive treatment with antenatal and possibly postnatal steroids, surfactant administration, respiratory support with ventilation, and a longer duration of oxygen exposure than a child born at 32 weeks gestation who may require relatively minimal therapy.

The studies tracking lung function over time imply ongoing and potentially progressive chronic lung disease throughout life. However, they do not elucidate if poor long-term lung health outcomes are linked to specific, remediable risk factors and do not allow at-risk individuals to be identified. A sophisticated life-course approach would be beneficial to understand the impact of preterm birth and try an elucidate how each risk factor directly impacts on long term lung health to ensure that neonatal care and ongoing follow up is further optimized and appropriately targeted (135, 136).

## The Effects of Moderate and Late Preterm Birth and Early Term Birth on Respiratory Outcomes

Much of this review focuses on the effects of very and extremely preterm infants, however there is growing interest in the long term respiratory outcomes of moderate preterm (32–33 weeks gestation), late preterm (34–36 weeks gestation), and early term born children (37–38 weeks gestation) in the current era of neonatal care. Late preterm and early term births account for between 3 to 6% and 15 to 30% respectively across different countries, with higher rates in high income countries (137). It has been recognized that infants born in the late preterm and early term periods have increased respiratory morbidity during the neonatal period, but there is also emerging evidence that this group of children are also at higher risk for developing respiratory symptoms such as cough and wheeze, and that they are at greater risk of lower respiratory viral infections (138–140). There is also evidence that children born moderate-late preterm also have reduced lung function compared with term born children, and there is conflicting evidence regarding whether or not children born moderate-late preterm may have a period of “catch up” growth to reach lung function levels of those born at term (15, 138). Unfortunately, there is very limited longitudinal lung function data on this cohort, making it difficult to track lung function trajectories in these children to predict future outcomes, or to identify factors which may increase their risk of adverse respiratory outcomes later in life. Given these births account for a significant proportion of global births, there is much value in future studies which focus on this area.

## Conclusion

There is increasing evidence that children and young people born pre-term may follow a number of different postnatal lung function trajectories. In addition, there are numerous factors which have the potential to modify which trajectory is followed. There are many antenatal, neonatal, and postnatal factors which may influence lung function trajectory, however more research is required to determine how these interact, and whether there are interventions that may improve outcomes.

## Author Contributions

All authors contributed to the literature review, intellectual input, drafting and final review of the manuscript.

## Conflict of Interest

The authors declare that the research was conducted in the absence of any commercial or financial relationships that could be construed as a potential conflict of interest.
